# Supplementary material for: The Dark Side of Healthy Eating: Links between Orthorexic Eating and Mental Health
Source: Nutrients. 2020 Nov 28;12(12):3662. doi: 10.3390/nu12123662 (PMC7761061; doi:10.3390/nu12123662)
Supplement: Supplementary file 1 [file nutrients-12-03662-s001.pdf]

**Supplementary material for: Strahler (nutrients-1013039)** ***The Dark Side of Healthy Eating: Links between Orthorexic Eating and Mental Health***

**Table S1.** Multiple regression analyses predicting wellbeing, life satisfaction and stress by healthy orthorexia (TOS-HeOr) and orthorexia nervosa (TOS-OrNe).

| | WHO-5 | | | L1 | | | PSS-10 | | |
|---|---|---|---|---|---|---|---|---|---|
| | **b** | **SE b** | **β** | **b** | **SE b** | **β** | **b** | **SE b** | **β** |
| Block 1 | | | | | | | | | |
| constant | 60.52 | 1.31 | | 7.31 | 0.12 | | 14.23 | 0.45 | |
| gender# | -4.77 | 1.53 | -0.11** | -0.21 | 0.14 | -0.06 | 3.00 | 0.53 | 0.20*** |
| Block 2 | | | | | | | | | |
| constant | 60.62 | 1.23 | | 7.31 | 0.11 | | 14.31 | 0.42 | |
| gender | -4.90 | 1.43 | -0.11** | -0.20 | 0.13 | -0.05 | 2.88 | 0.49 | 0.19*** |
| TOS-HeOr | 1.43 | 0.15 | 0.38*** | 0.09 | 0.01 | 0.26*** | -0.37 | 0.05 | -0.28*** |
| TOS-OrNe | -1.59 | 0.18 | -0.34*** | -0.14 | 0.02 | -0.33*** | 0.68 | 0.06 | 0.41*** |
| Block 3 | | | | | | | | | |
| constant | 59.81 | 1.26 | | 7.22 | 0.12 | | 14.60 | 0.43 | |
| gender | -4.88 | 1.43 | -0.11** | -0.19 | 0.13 | -0.05 | 2.88 | 0.49 | 0.19*** |
| TOS-HeOr | 1.46 | 0.15 | 0.38*** | 0.09 | 0.01 | 0.27*** | -0.38 | 0.05 | -0.28*** |
| TOS-OrNe | -1.88 | 0.21 | -0.40*** | -0.17 | 0.02 | -0.41*** | 0.78 | 0.07 | 0.47*** |
| Interaction | 0.08 | 0.03 | 0.11** | 0.01 | <0.01 | 0.13** | -0.03 | 0.01 | -0.11** |

#0=male, 1=female; WHO-5, Well-being Index; L1, Life satisfaction Scale; PSS-10, Perceived Stress Scale. WHO-5 model: $R^2$ =.012 for Block 1, $\Delta R^2$=.131 for Block 2, $\Delta R^2$=.008 for Block 3 ($p_{block1}$=0.002, $p_{block2}$<0.001, $p_{block3}$=0.009). L1 model: $R^2$ =.003 for Block 1, $\Delta R^2$=.093 for Block 2, $\Delta R^2$=.012 for Block 3 ($p_{block1}$=0.127, $p_{block2}$<0.001, $p_{block3}$=0.002). PSS-10 model: $R^2$ =.040 for Block 1, $\Delta R^2$=.134 for Block 2, $\Delta R^2$=.008 for Block 3 ($p_{block1}$<0.001, $p_{block2}$<0.001, $p_{block3}$=0.006). *p<0.05, **p<0.01, ***p<0.001.

**Table S2.** Multiple regression analyses predicting anxiety, depression and stress by healthy orthorexia (TOS-HeOr) and orthorexia nervosa (TOS-OrNe).

| | **HADS-A** | | | **HADS-D** | | | **DASS21-D** | | | **DASS21-A** | | | **DASS21-S** | | |
|---|---|---|---|---|---|---|---|---|---|---|---|---|---|---|---|
| | **b** | **SE b** | **β** | **b** | **SE b** | **β** | **b** | **SE b** | **β** | **b** | **SE b** | **β** | **b** | **SE b** | **β** |
| Block 1 | | | | | | | | | | | | | | | |
| constant | 6.67 | 0.43 | | 5.23 | 0.42 | | 2.55 | 0.32 | | 1.27 | 0.22 | | 3.31 | 0.35 | |
| gender# | 0.60 | 0.48 | 0.06 | -0.91 | 0.47 | -0.10 | 0.78 | 0.40 | 0.10 | 0.74 | 0.27 | 0.14** | 1.74 | 0.43 | 0.20*** |
| Block 2 | | | | | | | | | | | | | | | |
| constant | 6.74 | 0.39 | | 5.17 | 0.39 | | 2.64 | 0.30 | | 1.33 | 0.21 | | 3.42 | 0.32 | |
| gender | 0.44 | 0.44 | 0.05 | -0.92 | 0.43 | -0.10* | 0.72 | 0.36 | 0.09* | 0.68 | 0.26 | 0.12** | 1.64 | 0.40 | 0.19*** |
| TOS-HeOr | -0.18 | 0.04 | -0.23*** | -0.26 | 0.04 | -0.35*** | -0.18 | 0.04 | -0.25*** | -0.08 | 0.03 | -0.15** | -0.15 | 0.04 | -0.18** |
| TOS-OrNe | 0.44 | 0.05 | 0.50*** | 0.35 | 0.05 | 0.41*** | 0.45 | 0.05 | 0.45*** | 0.26 | 0.04 | 0.38*** | 0.47 | 0.06 | 0.43*** |
| Block 3 | | | | | | | | | | | | | | | |
| constant | 6.82 | 0.40 | | 5.47 | 0.39 | | 2.82 | 0.31 | | 1.41 | 0.22 | | 3.56 | 0.34 | |
| gender | 0.44 | 0.44 | 0.05 | -0.94 | 0.43 | -0.10* | 0.71 | 0.36 | 0.09 | 0.68 | 0.26 | 0.12** | 1.64 | 0.40 | 0.19*** |
| TOS-HeOr | -0.18 | 0.04 | -0.24*** | -0.26 | 0.04 | -0.35*** | -0.19 | 0.04 | -0.26*** | -0.08 | 0.03 | -0.16** | -0.15 | 0.04 | -0.19*** |
| TOS-OrNe | 0.47 | 0.05 | 0.53*** | 0.44 | 0.05 | 0.52*** | 0.52 | 0.06 | 0.53*** | 0.30 | 0.04 | 0.43*** | 0.53 | 0.07 | 0.49*** |
| Interaction | -0.01 | 0.01 | -0.05 | -0.03 | 0.01 | -0.20*** | -0.02 | 0.01 | -0.12* | -0.01 | 0.01 | -0.08 | -0.02 | 0.01 | -0.09 |

#0=male, 1=female; HADS, Hospital Anxiety (A) and Depression (D) Scale; DASS21, Depression (D), Anxiety (A) and Stress (S) Scale. HADS-A model: $R^2$ =.004 for Block 1, $\Delta R^2$=.185 for Block 2, $\Delta R^2$=.002 for Block 3 ($p_{block1}$=0.218, $p_{block2}$<0.001, $p_{block3}$=0.358). HADS-D model: $R^2$ =.010 for Block 1, $\Delta R^2$=.143 for Block 2, $\Delta R^2$=.027 for Block 3 ($p_{block1}$=0.051, $p_{block2}$<0.001, $p_{block3}$<0.001). DASS21-D model: $R^2$ =.010 for Step1, $\Delta R^2$=.161 for Step 2, $\Delta R^2$=.009 for Step 3 ($p_{block1}$=0.049, $p_{block2}$<0.001, $p_{block3}$=0.037). DASS21-A model: $R^2$ =.018 for Block 1, $\Delta R^2$=.115 for Block 2, $\Delta R^2$=.004 for Block 3 ($p_{block1}$=0.007, $p_{block2}$<0.001, $p_{block3}$=0.192). DASS21-S model: $R^2$ =.040 for Block 1, $\Delta R^2$=.146 for Block 2, $\Delta R^2$=.005 for Block 3 ($p_{block1}$<0.001, $p_{block2}$<0.001, $p_{block3}$=0.124). *p<0.05, **p<0.01, ***p<0.001.
